# Supplementary material for: Spatial Organisation and Invasive Behaviour of Metastatic Cutaneous Squamous Cell Carcinoma-Derived Multicellular Spheroids Reflect Tumour Cell Phenotype
Source: Cancers (Basel). 2025 Oct 22;17(21):3399. doi: 10.3390/cancers17213399 (PMC12608614; doi:10.3390/cancers17213399)
Supplement: Supplementary file 1 [file cancers-17-03399-s001.zip › cancers-3885994_Supplementary figures and tables.pdf]

Supplementary Materials

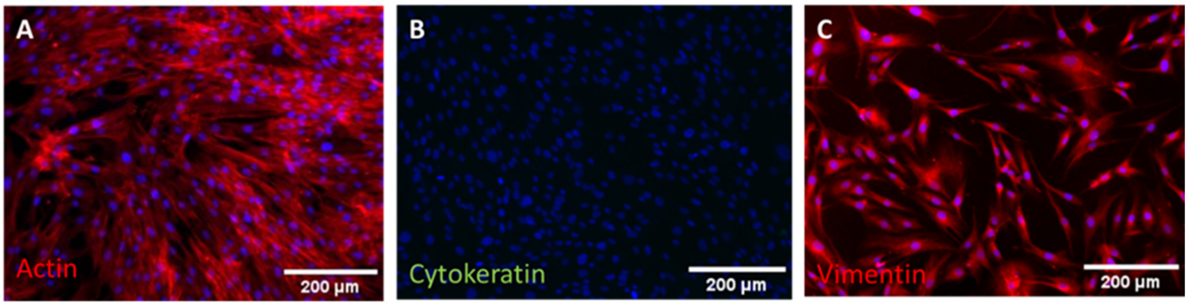

Supplementary Figure S1. Immunofluorescent staining of LNFs for (A) actin, (B) pan-cytokeratin, and (C) vimentin. LNFs displayed the marker profile as expected for a fibroblast cell line (i.e., cytokeratin negative and vimentin positive). An actin skeleton rich in contractile fibres (as opposed to a cortical actin ring observed in keratinocytes) confirms their identity.

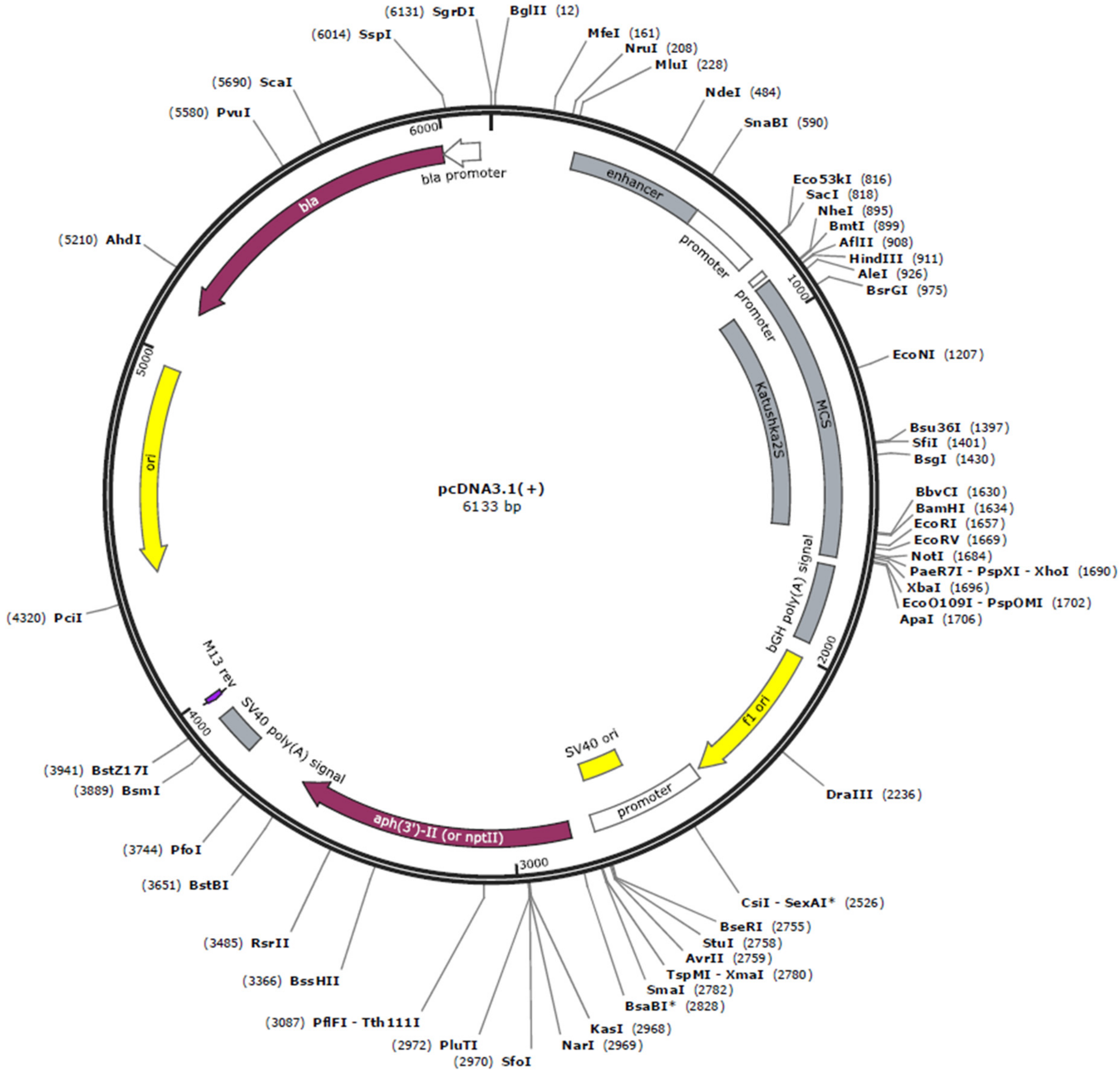

Supplementary Figure S2. Katushka2S plasmid map. Visualised using SnapGene software.

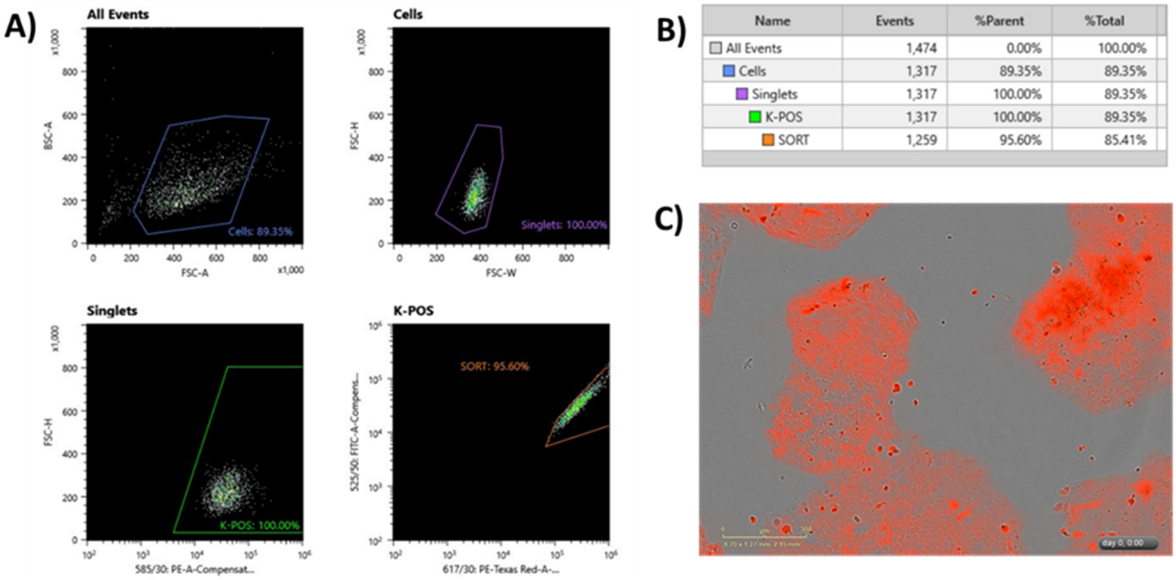

Name	Events	%Parent	%Total
All Events	1,474	0.00%	100.00%
Cells	1,317	89.35%	89.35%
Singlets	1,317	100.00%	89.35%
K-POS	1,317	100.00%	89.35%
SORT	1,259	95.60%	85.41%

Supplementary Figure S3. Representative gating strategy, purity report, and image of Katushka2S-transfected UW-CSCC1 cells. Cells were gated for viable cells, single cells, and fluorescence intensity (Panel A). This resulted in completely transfected cell population with very high proportions of very bright cells (Panels A and B). These results were confirmed using IncuCyte enabling fluorescence of Katushka2S expressing cells (red) (Panel C).

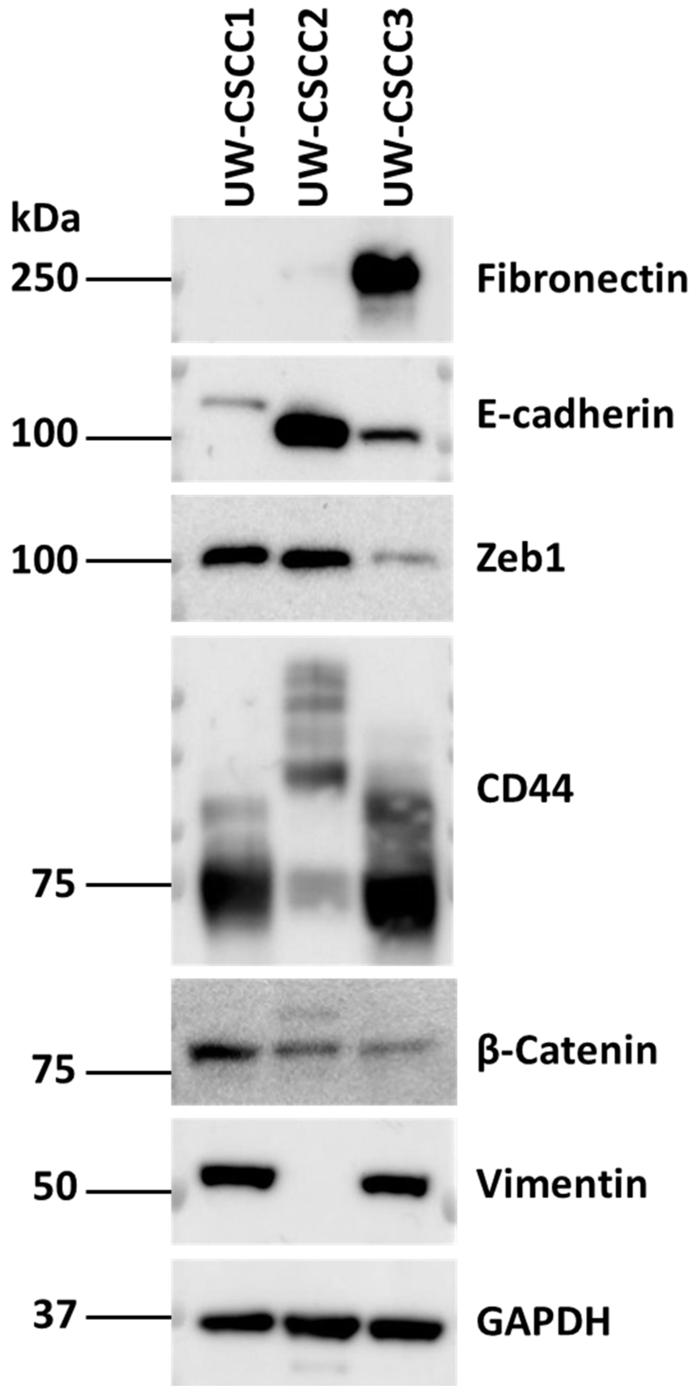

Supplementary Figure S4. Protein expression of EMT markers in metastatic cSCC cell lines. Baseline protein expression levels of UW-CSCC1, UW-CSCC2, and UW-CSCC3 (Supplementary Figure S5) in monolayer culture under standard culture conditions. Whole cell lysates were analysed via SDS-PAGE and Western blot. Refer to Supplementary Table S4 for antibodies and dilutions used.

A)

**Intermediary filaments**	Vimentin and cytokeratin positive
**Cell adhesion molecules**	Moderate E-cadherin (*CDH1)*
**EMT transcription factors**	Expression of EMT-TFs
**Morphology**	Pyramidal, elongated shape
	**Intermediate/Mesenchymal**

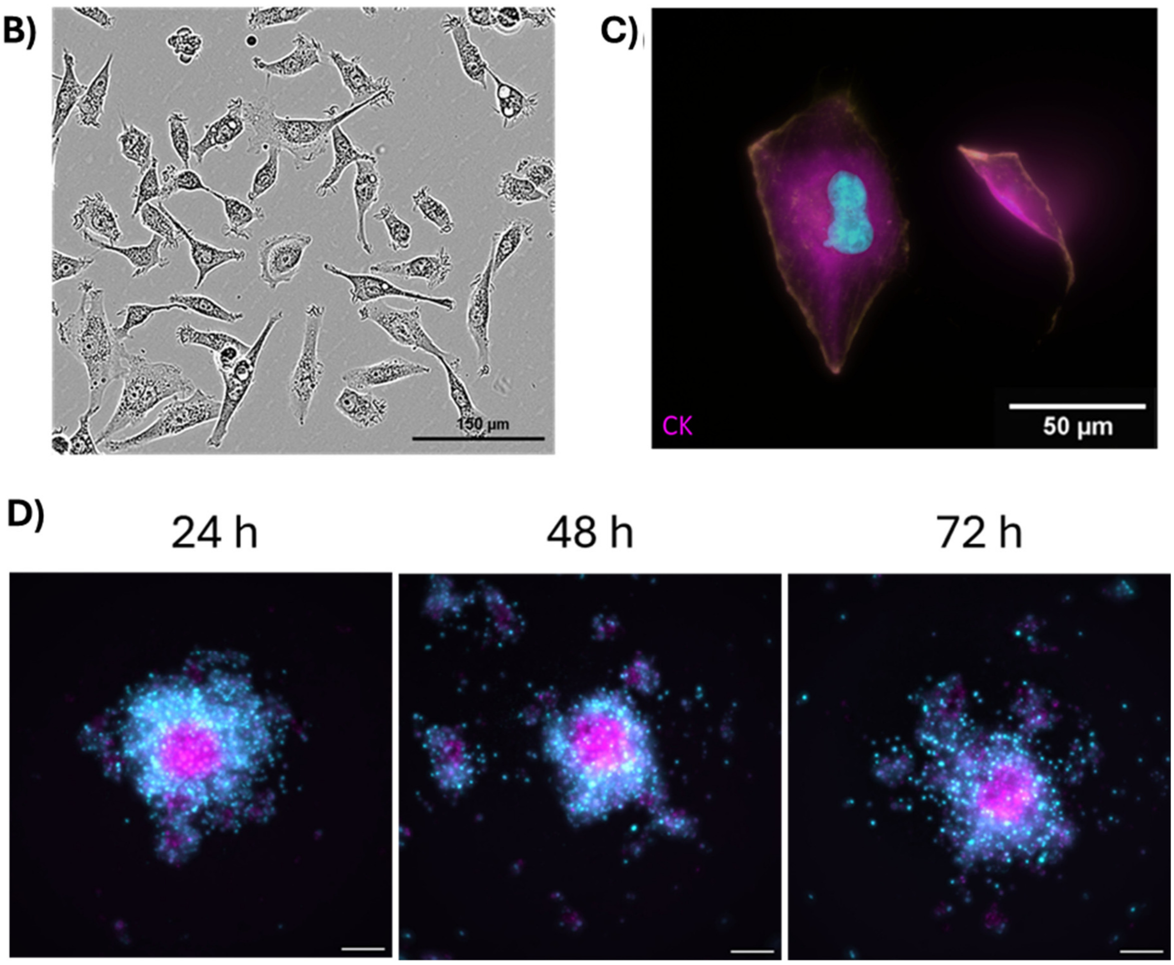

Supplementary Figure S5. Novel metastatic cSCC cell line UW-CSCC3 with intermediate EMT phenotype forms intermediate MCTS with DFs. Cell line was established and its epithelial origin and tumorigenicity confirmed as described previously [25]. A) EMT phenotype summary for UW-CSCC3, based on protein and transcriptomic data (Supplementary Table S4, Supplementary Table S3). B) Morphology of UW-CSCC3, taken during routine cell culture. C) Staining for cytokeratin (magenta), with nuclei and actin cytoskeleton counterstained in cyan and yellow respectively. D) UW-CSCC3/DF MCTS spatial architecture. UW-CSCC3 cells (cyan) and DFs (magenta) were stained with CellTracker™

Green and Red, respectively, and imaged every 24 hours post-seeding to assess spheroid formation and spatial architecture. Imaged using DMi8 microscope (Leica, Germany). Scale bar = 150 µm.

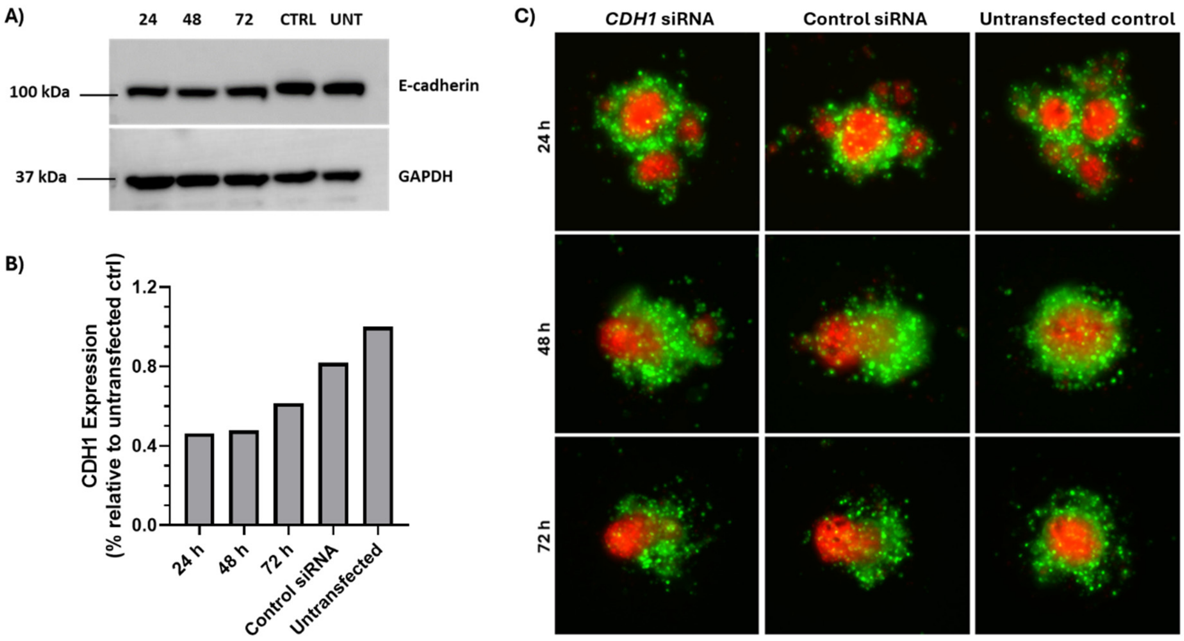

Supplementary Figure S6. CDH1 siRNA knockdown of UW-CSCC2 via reverse transfection. A) Western blots depicting E-cadherin and GAPDH expression for UW-CSCC2 cells reverse transfected with 150 pmol siRNA and 6 µL RNAiMAX for 24, 48, and 72 h, alongside control siRNA (CTRL) and untransfected (UNT) CSCC2 control cells. B) Densitometry quantification of CDH1 knockdown. E-cadherin expression was normalised to untransfected baseline E-cadherin expression. C) Representative images of MCTS architecture following UW-CSCC2 CDH1 knockdown. CellTrackerTM stained MCTS comprised of UW-CSCC2 (green)/DFs (red) were imaged using a Leica DMi8 microscope (20 × objective), over 24, 48, and 72 h. MCTS were seeded in quintuplicate. See Supplementary Methods for details.

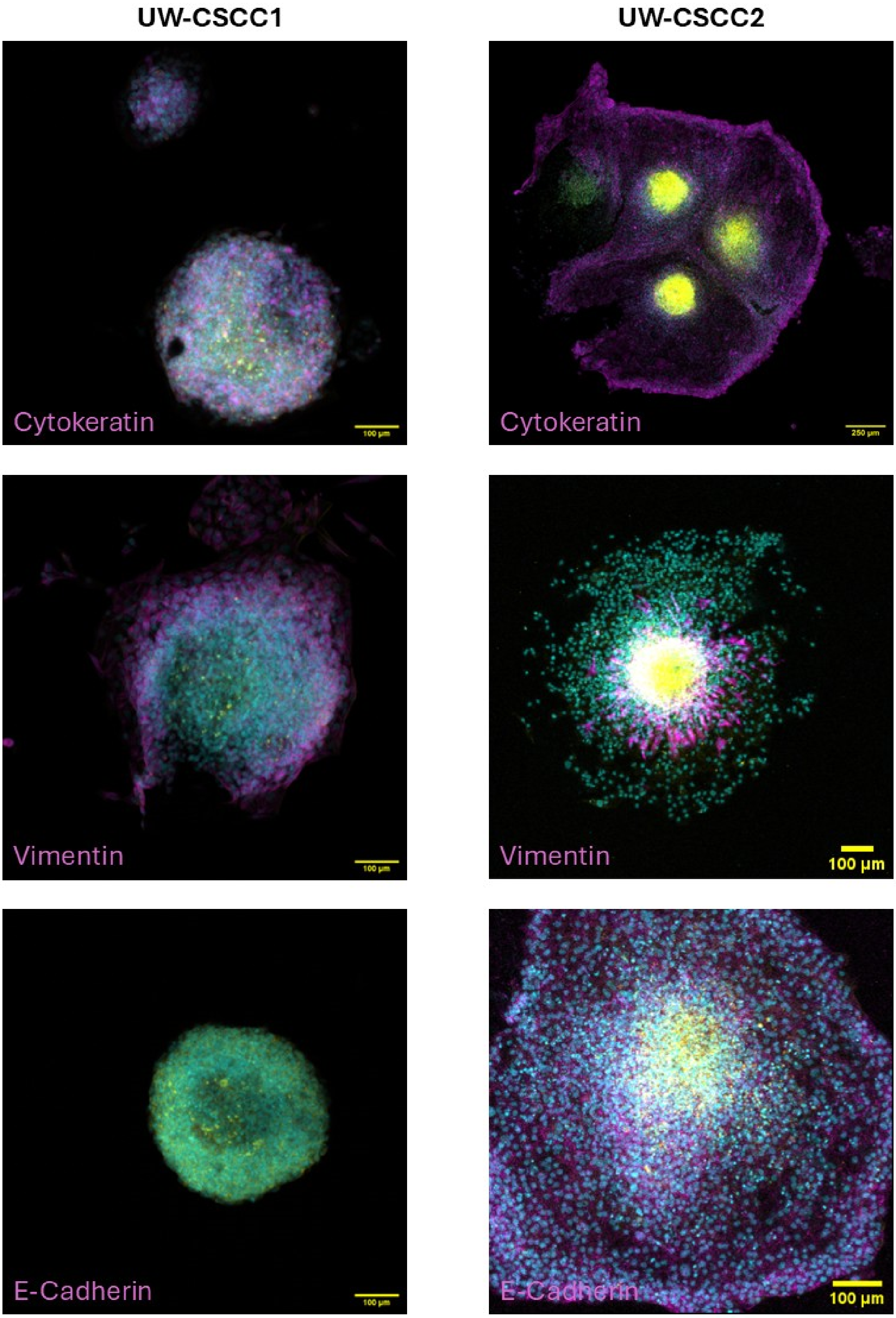

Supplementary Figure S7. Vimentin, E-cadherin and cytokeratin expression in non-embedded DF MCTS. Non-embedded UW-CSCC1/DF and UW-CSCC2/DF MCTS were stained for cytokeratin, E-cadherin and vimentin (magenta). Nuclei (cyan) and actin cytoskeleton (yellow) were counterstained with Hoechst-33342 and ActinRed™ 555 respectively. Scale bars indicated within each image.

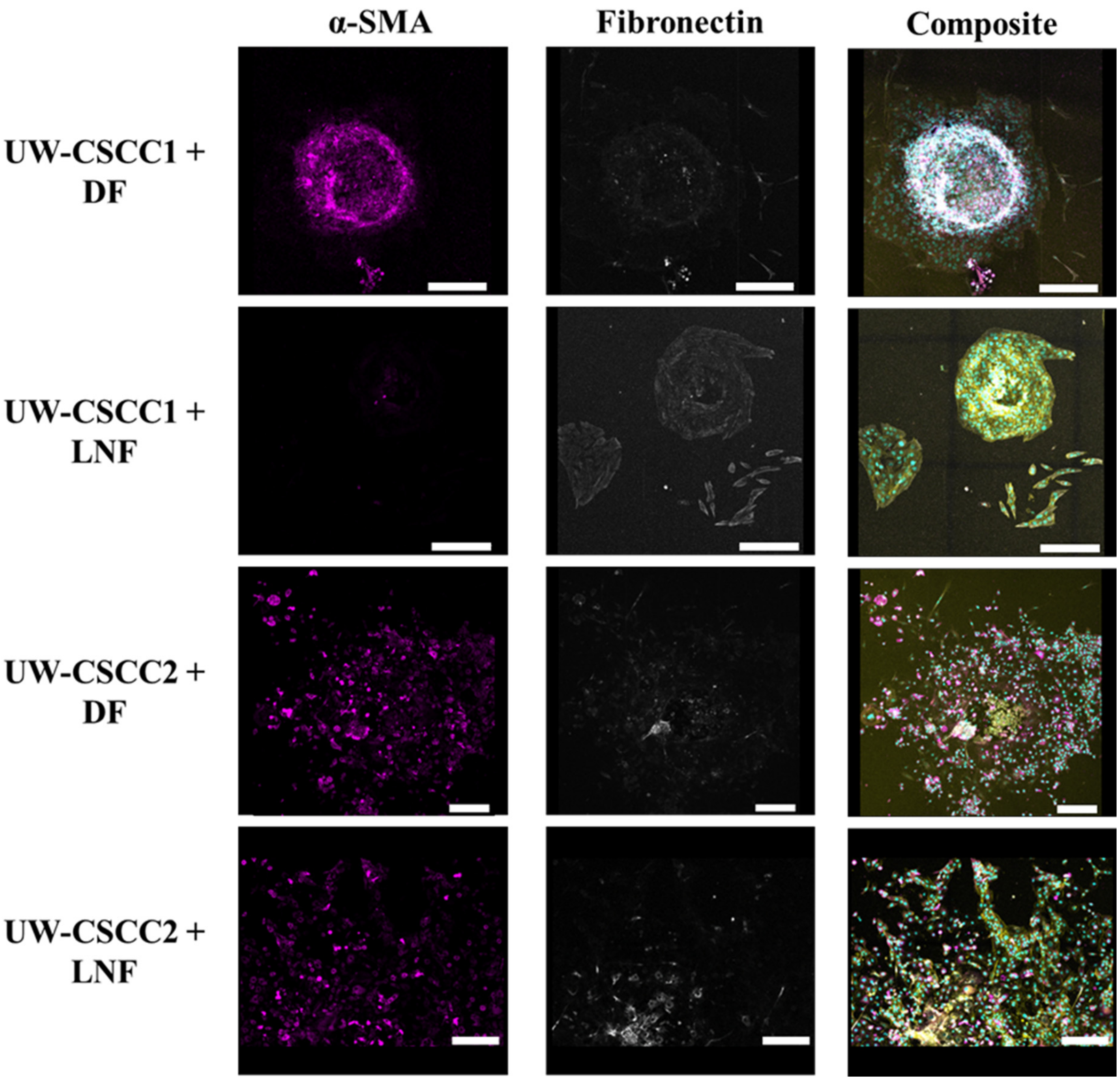

Supplementary Figure S8. α-SMA (ACTA2) and fibronectin (FN1) expression in collagen-embedded skin MCTS. UW-CSCC1 or UW CSCC2 and either DFs or LNFs were seeded in ultra-low attachment plates and the resulting MCTS were embedded in collagen I. After 80h of invasion, MCTS were stained for α-SMA (magenta) and fibronectin (grey). Additionally, the nuclei (cyan) and actin cytoskeleton (yellow) were stained with Hoechst 33342 and ActinRed™ 555, respectively. The images represent the summation of all slices of lightening processed images. Brightness and contrast were adjusted for presentation purposes. The scale bar represents 250 µm.

Supplementary Table S1. Composition of cell culture media for routine cell culture and MCTS generation.

Cell line(s)	Cell culture media
**UW-CSCC1 or LNFs or DFs**	DMEM + 10% FCS, glucose (4,500 mg/L), P/S
**UW-CSCC2**	Advanced DMEM/F12 + 2% FCS, L-Glu (20 mM), P/S, EGF (20 ng/mL)
**UW-CSCC1 + LNFs/DFs**	DMEM + 10% FCS, glucose (4,500 mg/L), P/S
**UW-CSCC2 + LNFs/DFs**	DMEM + 10% FCS, glucose (4,500 mg/L), P/S, EGF (20 ng/mL)
**UW-CSCC3-M + DFs**	DMEM + 10% FCS, glucose (4,500 mg/L), P/S

Dulbecco's Modified Eagle Medium, DMEM; foetal calf serum, FCS; lymph node fibroblasts, LNF; dermal fibroblasts (DFs); penicillin/streptomycin (50 U/mL), P/S; L-Glutamine, L-Glu; Epidermal growth factor, EGF.

Supplementary Table S2. Antibodies, stains, and dilutions for immunocytochemistry.

Antigen/Stain	Host	Dilution	Manufacturer (# Catalogue)
Vimentin	Rb	1:1000	Abcam (ab92547)
Pan-Cytokeratin	Ms	1:1000	Sigma Aldrich (C2562)
E-Cadherin	Ms	1:1000	Abcam (ab76055)
N-Cadherin	Ms	1:1000	Sigma Aldrich (C3865)
α – Smooth muscle actin	Ms	1:1000	Abcam (ab7817)
Fibronectin	Rb	1:1000	Abcam (ab32419)
Anti-ms IgG (AF488)	Dk	1:1000	Abcam (ab150105)
Anti-rb IgG (AF647)	Gt	1:1000	Abcam (ab150079)
ActinRed™ 555 ReadyProbes™ Reagent (Rhodamine phalloidin)	-	2 drops/mL	ThermoFisher Scientific (R37112)
Hoechst 33342 (10 mg/mL)	-	1:2500	ThermoFisher Scientific (H3570)

Ms, mouse; Rb, Rabbit; Dk, Donkey; Gt, Goat; AF, AlexaFluor.

Supplementary Table S3. Transcripts per million (TPM) counts of EMT transcription factors in metastatic cSCC cell lines. RNA-Seq data obtained and analysed as previously described [25]. TF: transcription factor.

Gene	Function	UW-CSCC1	UW-CSCC2	UW-CSCC3
TWIST1	EMT TF	21.88	6.01	17.71
TWIST2	EMT TF	4.89	6.67	11.08
SNAI1	EMT TF	24.87	1.34	2.51
SNAI2	EMT TF	40.61	31.31	57.27

ZEB1	EMT TF	15.01	0.80	15.51
ZEB2	EMT TF	48.70	0.75	28.37
OVOL1	Anti-EMT TF	0.01	10.26	0.02
OVOL2	Anti-EMT TF	0.06	4.46	0

Supplementary Table S4. Antibodies and dilutions used for Western blot visualisation of EMT marker protein expression.

**Primary Antibodies**			
**Antibody**	**Host**	**Dilution**	**Manufacturer (Catalogue Number)**
Vimentin	Rb	1:2000	Abcam (ab92547)
E-cadherin	Ms	1:2000	Abcam (ab76055)
CD44	Rb	1:2000	Cell Signalling Technology (37259T)
β-catenin	Rb	1:2000	Cell Signalling Technology (8480T)
EpCAM	Ms	1:1000	Abcam (ab7504)
Zeb1	Rb	1:1000	Abcam (ab276129)
Fibronectin	Rb	1:1000	Abcam (ab32419)
SLUG	Rb	1:1000	Abcam (ab27568)
GAPDH	Ms	1:10000	Sigma Aldrich (G8795)
**Secondary Antibodies**			
**Antibody**	**Host**	**Dilution**	**Manufacturer (Catalogue Number)**
Anti-Rabbit IgG, HRP-linked Goat	Gt	1:5000	Cell Signalling Technology (7074)
Anti-Mouse IgG, HRP-linked Goat	Gt	1:5000	Abcam (ab205719)

Rb: rabbit. Ms: mouse. Gt: goat.

Supplementary Methods

CDH1 siRNA knockdown of UW-CSCC2/DF MCTS

E-cadherin expressing UW-CSCC2 cells and DFs were collected and stained with 10 μM CellTracker™ Green and CellTracker™ Red respectively for 1 hour at 37°C. Stained UW-CSCC2 cells were reverse transfected with *CDH1* or Control siRNA (150 pmol; details below) using Lipofectamine™ RNAiMax Transfection Reagent (Invitrogen, 13778150) (6 μL) for 16 hours as per manufacturers' instructions. MCTS were formed in ultra-low attachment 96-well plates (1500 UW-CSCC2 + 1500 DFs per well) and MCTS architecture monitored via inverted light microscopy (Leica DMi8) over 72 hours.

***CDH1* siRNA:** *Silencer*™ Select CDH1 siRNA (Invitrogen, 4392420)

Control siRNA: *Silencer*™ Select Negative Control No.1 siRNA (Invitrogen, 4390843)
