# Supplementary material for: Spatial Organisation and Invasive Behaviour of Metastatic Cutaneous Squamous Cell Carcinoma-Derived Multicellular Spheroids Reflect Tumour Cell Phenotype
Source: Cancers (Basel). 2025 Oct 22;17(21):3399. doi: 10.3390/cancers17213399 (PMC12608614; doi:10.3390/cancers17213399)
Supplement: Supplementary file 1 [file cancers-17-03399-s001.zip › Raw Western Blots.pdf]

Raw Western blot images from Supplementary Figure S4

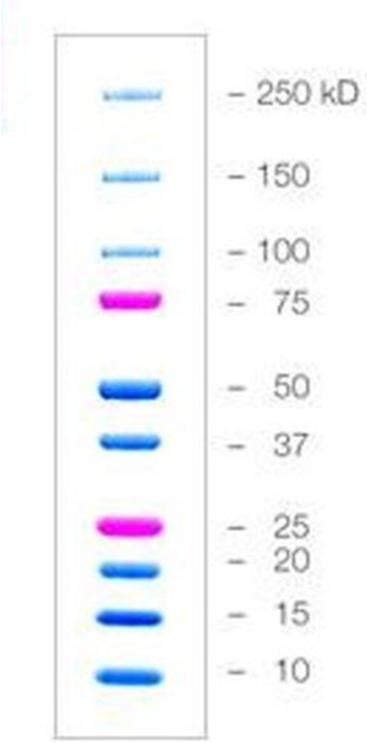

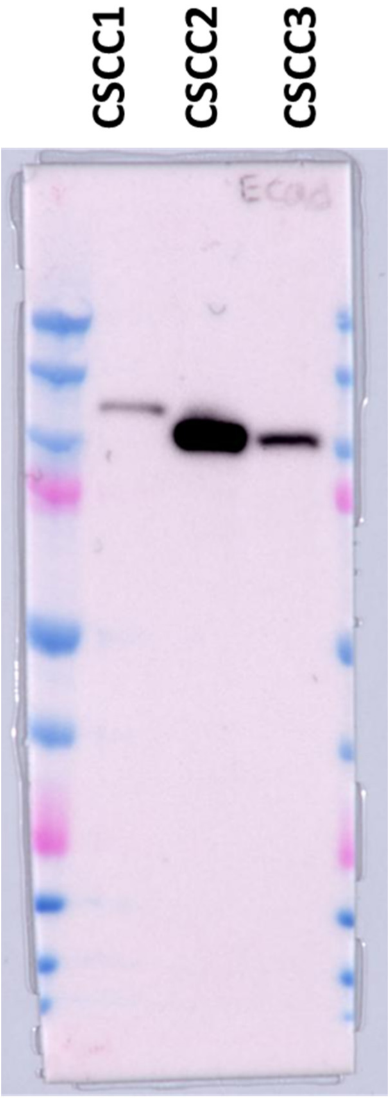

E-cadherin

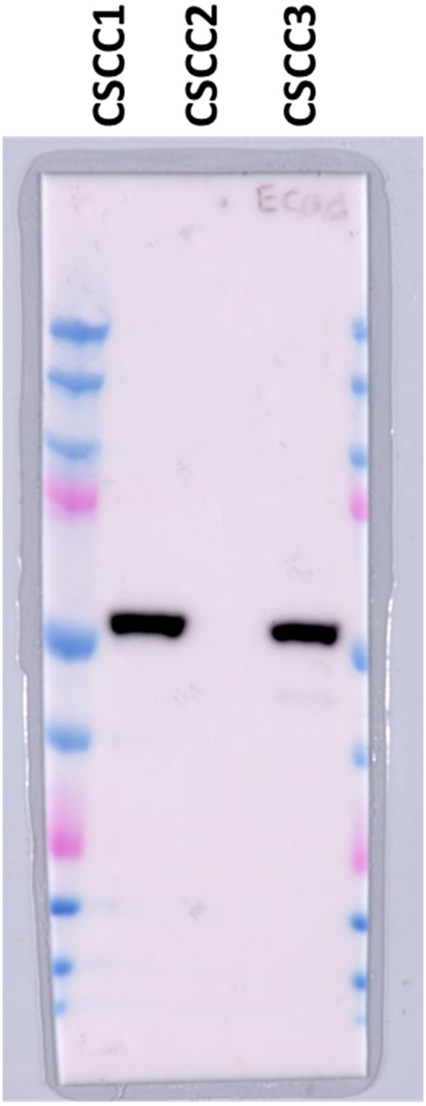

Vimentin

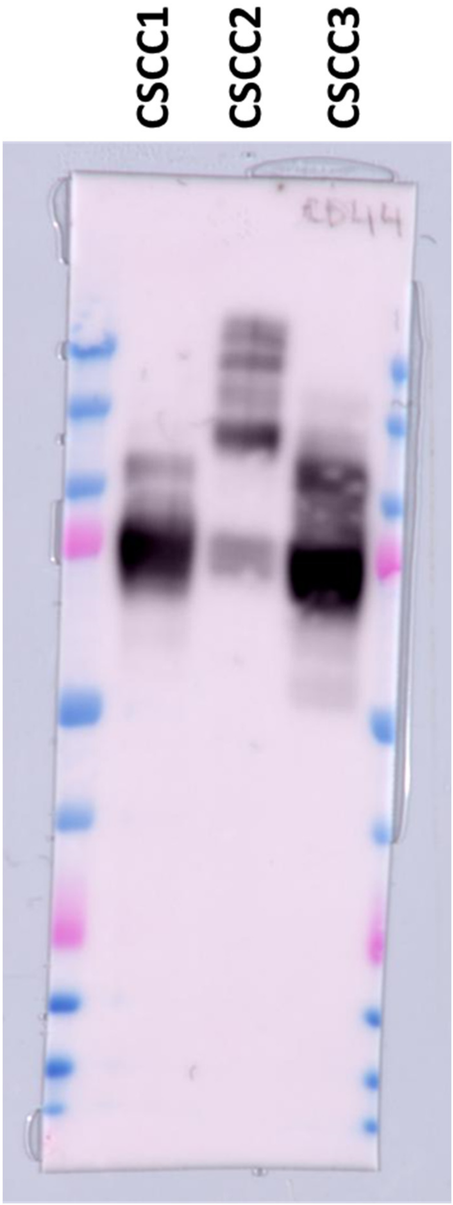

CD44

CSCC1 CSCC2 CSCC3

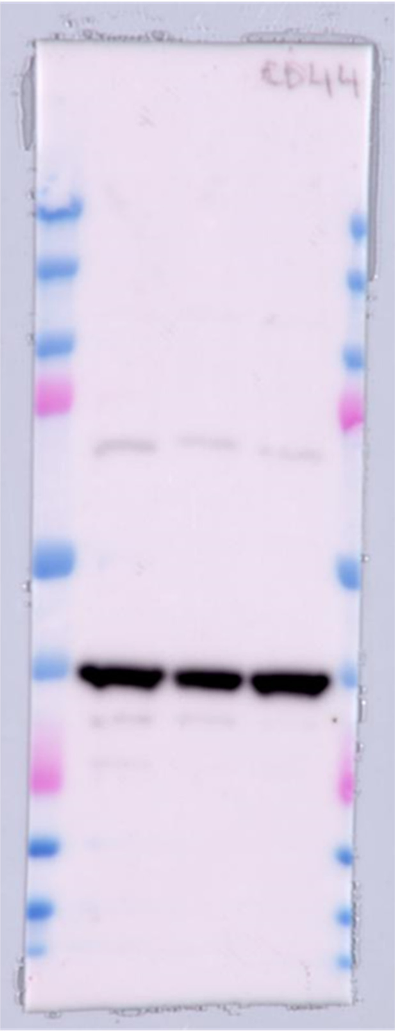

GAPDH

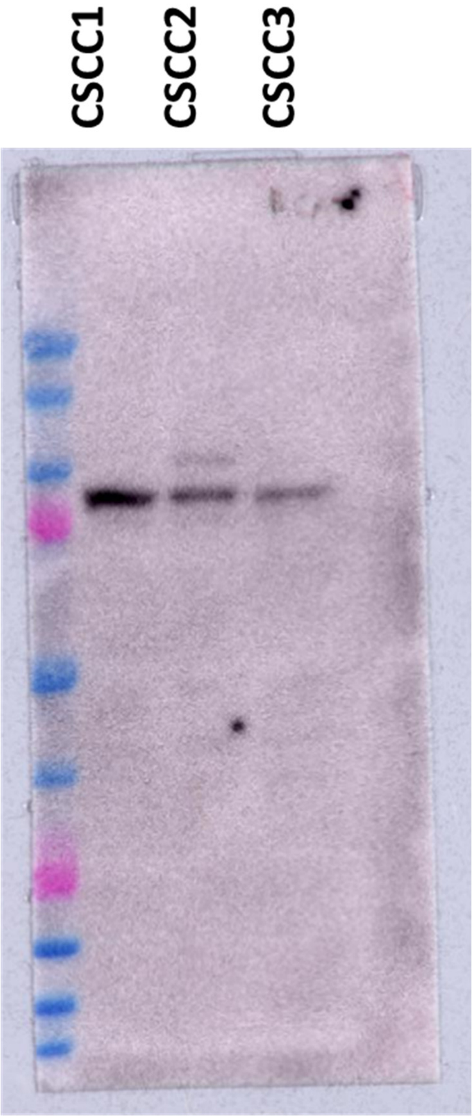

β-Catenin

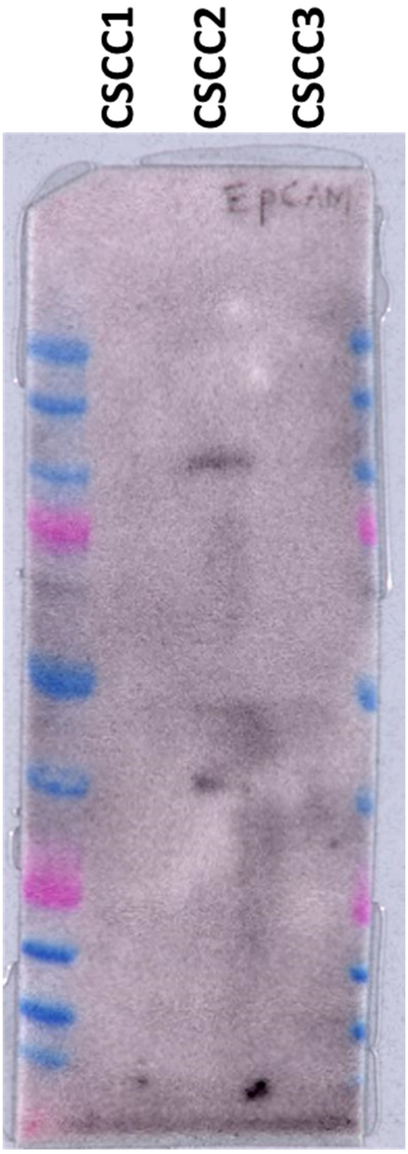

EpCAM

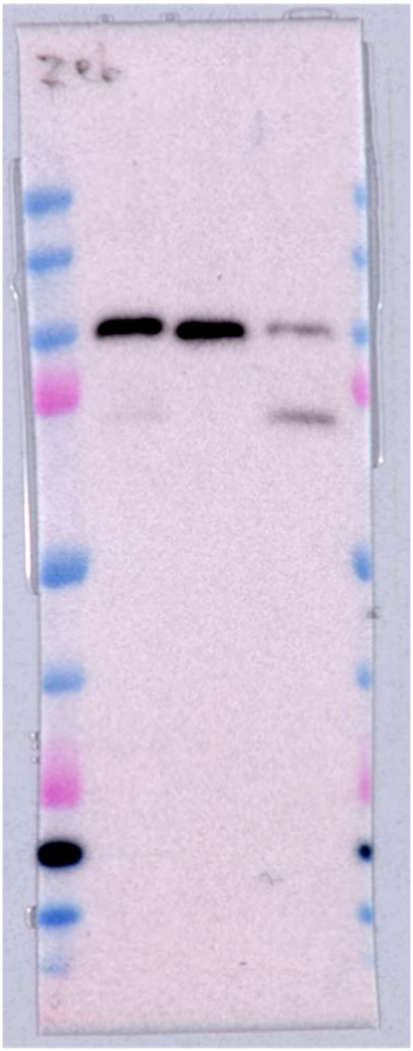

Zeb1

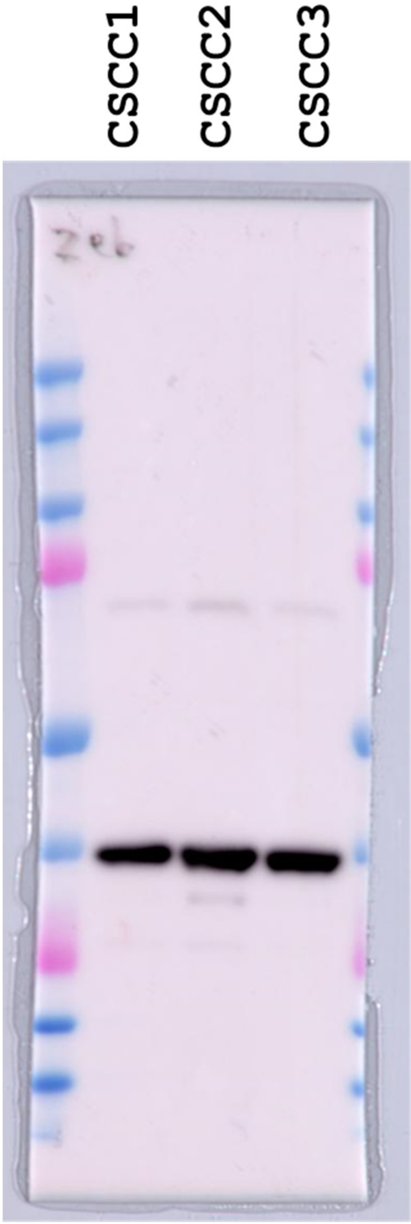

GAPDH

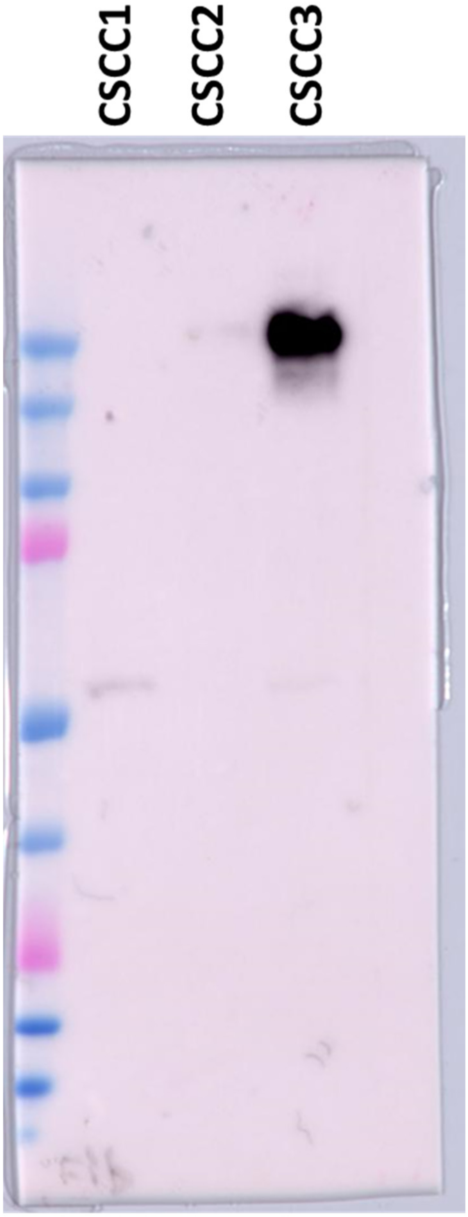

Fibronectin

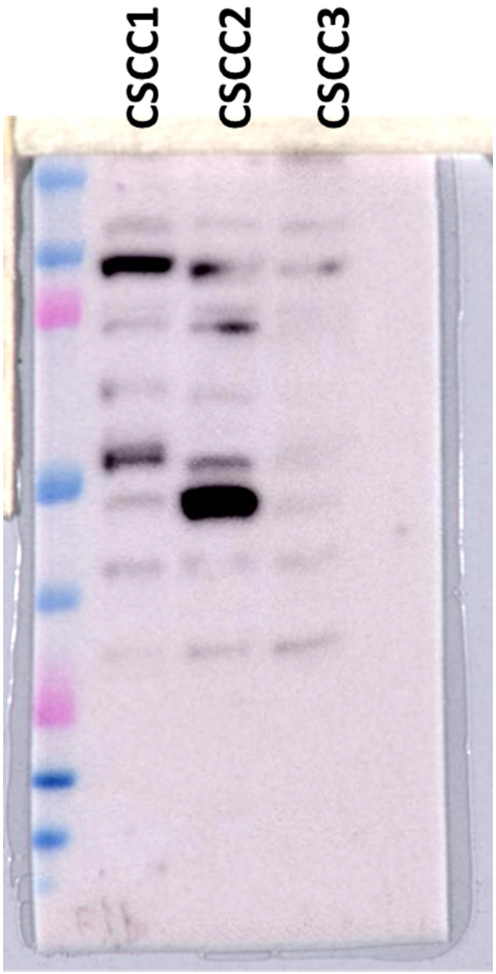

SLUG

Raw Western blot images from Supplementary Figure S6

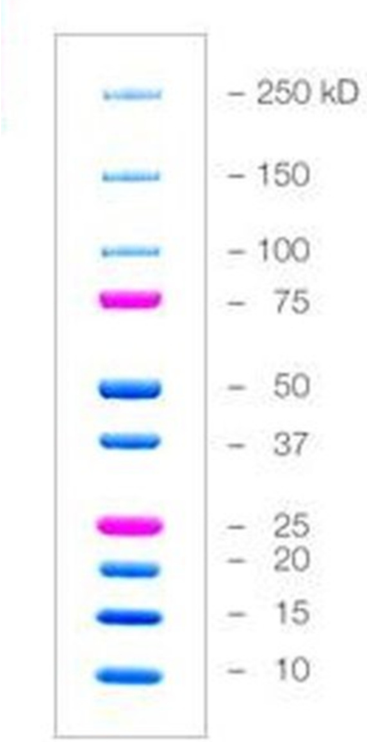

24h CDH1 KD
48h CDH1 KD
72h CDH1 KD
Ctrl siRNA (CTRL)
Untransfected control

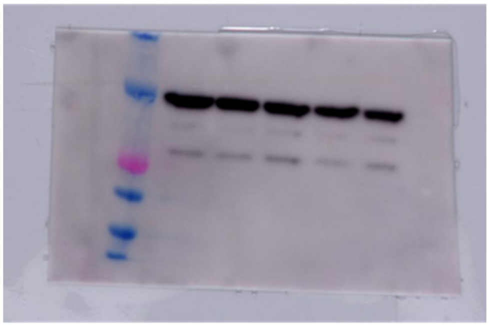

GAPDH

24h CDH1 KD
48h CDH1 KD
72h CDH1 KD
Ctrl siRNA (CTRL)
Untransfected control

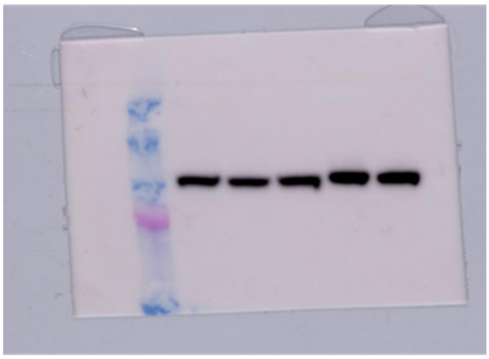

E-cadherin
