# Supplementary figures and images for: Spatial Organisation and Invasive Behaviour of Metastatic Cutaneous Squamous Cell Carcinoma-Derived Multicellular Spheroids Reflect Tumour Cell Phenotype
Source: Cancers (Basel). 2025 Oct 22;17(21):3399. doi: 10.3390/cancers17213399 (PMC12608614; doi:10.3390/cancers17213399)

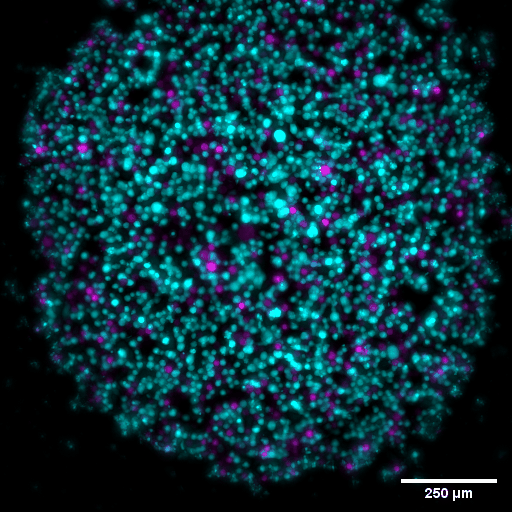

Supplement: Supplementary file 1 [file cancers-17-03399-s001.zip › Video S1._Live-cell_UW-CSCC1_and_DFs.gif]

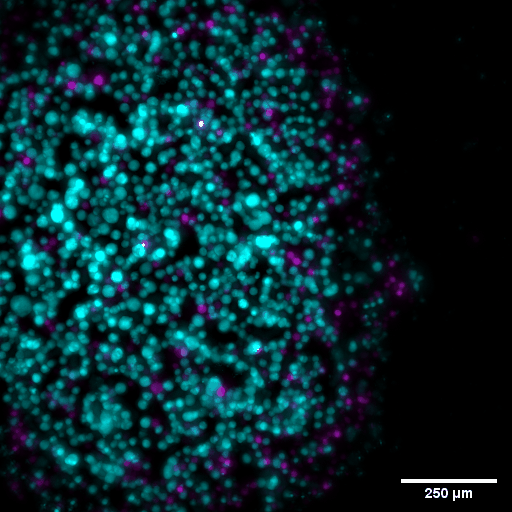

Supplement: Supplementary file 1 [file cancers-17-03399-s001.zip › Video S2. Live-cell_UW-CSCC1_and_LNFs.gif]

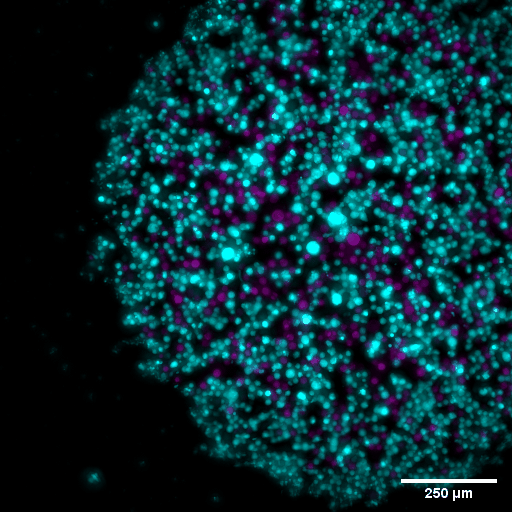

Supplement: Supplementary file 1 [file cancers-17-03399-s001.zip › Video S3. Live-cell_UW-CSCC2_and_DFs.gif]

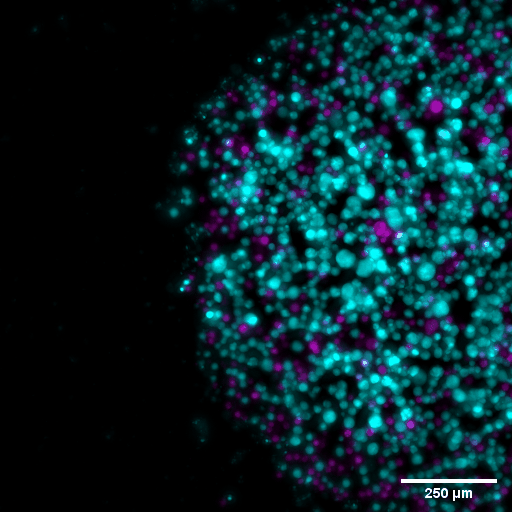

Supplement: Supplementary file 1 [file cancers-17-03399-s001.zip › Video S4. Live-cell_UW-CSCC2_and_LNFs.gif]
